# Bone-anchored titanium implants for auricular rehabilitation: case report and review of literature

**DOI:** 10.1590/S1516-31802009000300009

**Published:** 2009-10-06

**Authors:** Emne Hammoud Gumieiro, Luciano Lauria Dib, Ricardo Schmitutz Jahn, João Ferreira dos Santos, Ulf Nannmark, Gösta Granström, Márcio Abrahão

**Affiliations:** 1 MD. Postgraduate student, Department of Otorhinolaryngology and Head and Neck Surgery, Universidade Federal de São Paulo (Unifesp), São Paulo, Brazil.; 2 DDS, PhD. Professor, Department of Stomatology, Faculty of Dentistry, Universidade Paulista (Unip), São Paulo, Brazil.; 3 MD. Postgraduate student, Department of Otorhinolaryngology and Head and Neck Surgery, Universidade Federal de São Paulo (Unifesp); and Professor, Department of Periodontology, School of Dentistry, Santo Amaro University (Unisa), São Paulo, Brazil.; 4 MD. Postgraduate student, Department of Otorhinolaryngology and Head and Neck Surgery, Universidade Federal de São Paulo (Unifesp); and Professor, Department of Stomatology, School of Dentistry, Santo Amaro University (Unisa), São Paulo, Brazil.; 5 DDS, PhD. Professor, Department of Anatomy and Cell Biology, University of Gothenburg, Gothenburg, Sweden.; 6 DDS, MD, PhD. Professor, Department of Otolaryngology, Head and Neck Surgery, University of Gothenburg, Gothenburg, Sweden.; 7 MD, PhD. Professor, Department of Otorhinolaryngology and Head and Neck Surgery, Universidade Federal de São Paulo (Unifesp), São Paulo, Brazil.

**Keywords:** Ear deformities, acquired, Head and neck neoplasms, Hemangioma, Prostheses and implants, Outcome assessments (health care), Deformidades adquiridas da orelha, Neoplasias de cabeça e pescoço, Hemangioma, Próteses e implantes, Avaliação de resultados (cuidados de saúde).

## Abstract

**CONTEXT AND OBJECTIVE:**

Osseointegrated implants have acquired an important role in the prosthetic rehabilitation of patients with craniofacial defects. The main indications are lack of local tissue for autogenous reconstruction, previous reconstruction failure and selection of this technique by the patient. This paper presents a clinical case and discusses indications and advantages of the osseointegrated implant technique for retention of auricular prostheses.

**TYPE OF STUDY:**

Case report, Universidade Federal de São Paulo (UNIFESP).

**METHODS:**

A female patient received three auricular implants after surgical resection of a hemangioma in her left ear. The time taken for osseointegration of the temporal bone was three months. After fabrication of the implant-retained auricular prosthesis, the patient was monitored for 12 months.

**RESULTS:**

The clinical parameters evaluated showed good postoperative healing, healthy peri-implant tissue, good hygiene and no loss of implants. Good hygiene combined with thin and immobile peri-implant soft tissues resulted in minimal complications. Craniofacial implant integration appears to be site-dependent; increasing age affects osseointegration in the temporal bone. The frequency of adverse skin reactions in peri-implant tissues is generally low.

**CONCLUSION:**

The surgical technique for rehabilitation using implant-retained auricular prostheses seems to be simple. It is associated with low rates of adverse skin reactions and long-term complications. Prostheses anchored by osseointegrated implants seem to provide better retention than do prostheses supported on spectacle frames, less risk of discoloration through the use of adhesives and better esthetic results than do prostheses anchored in the surgical cavity.

## RESUMO

**CONTEXTO E OBJETIVO:**

Os implantes osseointegrados adquiriram papel importante na reabilitação protética de pacientes com defeitos craniofaciais. As principais indicações são ausência local de tecidos para reconstrução autógena, falha anterior de reconstrução e opção do paciente. Este artigo apresenta um caso clínico e discute as indicações e vantagens da técnica de implantes osseointegrados para a retenção de próteses auriculares.

**TIPO DE ESTUDO E LOCAL:**

Relato de caso clínico, Universidade Federal de São Paulo (UNIFESP).

**MÉTODOS:**

Paciente do sexo feminino recebeu três implantes na região auricular após ressecção cirúrgica de hemangioma na orelha esquerda. O tempo de osseointegração no osso temporal foi de três meses. Após a confecção de prótese auricular implanto-suportada, a paciente foi observada por 12 meses.

**RESULTADOS:**

Os parâmetros clínicos analisados mostraram boa cicatrização pós-operatória, saúde dos tecidos adjacentes ao implante, boa higiene e nenhuma perda de implantes. A boa higiene combinada a pouca espessura e imobilidade dos tecidos moles perimplantares resulta em poucas complicações. A integração dos implantes craniofaciais parece variar conforme o local, e a idade avançada afeta a osseointegração no osso temporal. A frequência de reações adversas cutâneas nos tecidos perimplantares é geralmente baixa.

**CONCLUSÃO:**

A técnica cirúrgica para a reabilitação com próteses auriculares implanto-suportadas parece ser simples e está associada a baixos índices de reações cutâneas adversas e complicações no longo prazo. As próteses ancoradas por implantes osseointegrados parecem proporcionar melhor retenção do que as próteses suportadas nas armações de óculos, menor possibilidade de descoloração pelo uso de adesivos e melhores resultados estéticos do que as próteses ancoradas na cavidade cirúrgica.

## INTRODUCTION

Congenital or acquired absence of facial structures caused by malformation, cancer treatment surgery or trauma leads to functional deficits and enormous psychological strain, and therefore requires rehabilitation.^1^ Auricular reconstruction is a challenging task for surgeons since it is a field of facial plastic surgery in which a wide array of reconstructive options often must be considered.^2^

Surgical procedures to reconstruct these defects may sometimes even be hampered by vascular compromise due to surgical bed irradiation, the physical condition of a patient when multiple surgical procedures are required and patients’ esthetic expectations regarding what can be achieved by reconstructive surgical procedures.^3^ The amount of soft tissue and cartilaginous or osseous support available is sometimes insufficient for a reconstruction that is functional and esthetic.^4^ Moreover, conventional autologous grafts for auricular reconstruction may produce inconsistent results, and revision of failed grafts is often unsatisfactory.^5^

Prosthetics have become available and have been developed into functional and esthetic alternatives to plastic and reconstructive surgery. Since the introduction of percutaneous endosseous implants for use with bone conduction hearing aids in 1977, implants also have acquired an important role in the prosthetic rehabilitation of patients with craniofacial defects.^6^^,^^7^ Prosthetic reconstruction of these structures, using cranial implants, provide an alternative approach towards rehabilitating patients with severe auricular defects.^5^ This has become a viable option that can offers several advantages over traditional reconstructive techniques.^4^

Osseointegration biotechnology has revolutionized ear prosthetic retention, and the benefits of osseointegrated alloplastic ear reconstruction have been well documented. By using prostheses anchored on osseointegrated implants, firm retention of the prosthesis is obtained. It is generally agreed that such retention is more secure than the retention obtained by using conventional glues or prostheses anchored on spectacles or steel springs, or through the use of undercuts.

This paper presents a clinical case and discusses the indications and advantages of the osseointegrated implant technique for the retention of auricular prostheses, based on a review of the literature.

## CLINICAL CASE

**Figure 1.**
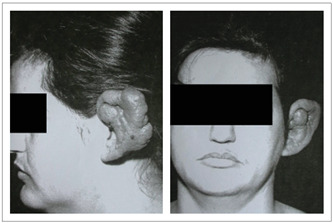
A 38-year-old female patient with a left-ear tumor diagnosed as hemangioma. Initial clinical appearance.

A 38-year-old female patient was treated surgically with total resection of her left ear, which presented a lesion diagnosed as hemangioma (Figure 1), and was subsequently indicated for ear replacement with an auricular prosthesis. Hemangiomas of the external ear are extremely rare entities that are readily treatable by means of surgical excision.^8^ This patient underwent tumor resection surgery at the Hospital A.C. Camargo in 1995.

In 2004, the patient was referred to the Center for Maxillofacial Rehabilitation of the Universidade Federal de São Paulo (Unifesp) for craniofacial rehabilitation with an extraoral implant-retained prosthesis. Informed consent was obtained from the patient with regard to publishing this paper.

### Surgical implant procedure and prosthetics

The patient received the implants in accordance with a two-stage surgical procedure developed at the University of Gothenburg, Sweden, which has a collaboration agreement with Unifesp’s Center for Maxillofacial Rehabilitation. Screw-shaped titanium implants are inserted into the temporal bone using a delicate surgical technique and, after the implants have healed in, it is possible to penetrate the skin to establish a reaction-free percutaneous passage.^9^^,^^10^^,^^11^^,^^12^ A gentle surgical technique is imperative in order not to damage the osteocytes, which might otherwise result in fibrous encapsulation instead of direct contact between implant and bone (osseointegration).^13^

During the first stage, three 3.75 mm x 4 mm implants (MasterExtra, Conexão, Sistema de Próteses, São Paulo, Brazil) were inserted into the bone surrounding the area with the craniofacial defect. After a previous computed tomography (CT) scan, coronal, axial and three-dimensional reconstruction images were used to measure the bone thickness in the mastoid region (at least 6 mm), and to investigate whether there might be any cellular bone areas, residual tumors or other lesions that could prevent the fixation of implants and interfere with the success of the surgical procedure. The patient went through the implant surgery under general anesthesia; 2 g of cephalexin was administered on a prophylactic basis. Surgical templates were used to assure optimal implant placement, adequate spacing and proper angulation. The available bone volume was also verified *in situ* at the time of surgery and was found to be sufficient for reliable insertion of the implants. A 4 mm longitudinal incision was made posterior to the external acoustic meatus and the temporal bone was exposed. The implants were handled with titanium-coated instruments and never directly by gloved hands, since even minute sterile contaminants on the surface of the implant might jeopardize osseointegration. The time taken for osseointegration was expected to be three months for implants inserted into the temporal bone.

The second stage consisted of thinning of the subcutaneous tissue, uncovering of the implants and attachment of abutments to the implants. This procedure included subcutaneous tissue reduction aimed at reducing the mobility between the implant and the skin. To facilitate cleaning, the skin needed to be devoid of hair follicles. One implant was kept buried. Healing caps were placed over the abutments and gauze soaked in ointment was wrapped around the healing caps to ensure good contact between the skin and the bone, and to prevent postoperative hematoma and swelling. The patient was released after recovery from general anesthesia. The postoperative management plan included oral analgesic prescription for few days and local hygiene instructions.

**Figure 2.**
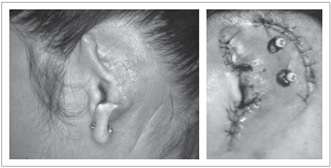
Auricular defect after resection surgery (left), Three craniofacial implants have been placed in the temporal region; one implant is buried (right).

The suture was removed after ten days and the patient did not complain of postoperative pain or complications during this period. The gauze dressings were changed weekly for a period of three weeks. Three to four weeks after the second stage, the healing was expected to have reached the point at which the prosthesis could be constructed and attached to the implants (Figure 2).

**Figure 3.**
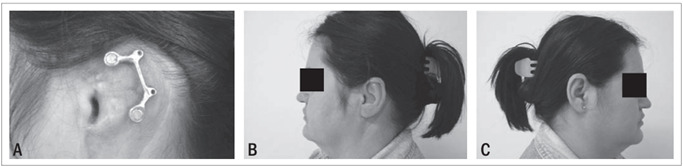
(A) Bar retention clip system (B) Implant-retained auricular prosthesis in situ. (C) Right ear.

Fabrication of the implant-retained prosthesis was started three weeks after abutment connection, which followed standard clinical and laboratory procedures.^14^ Retention was achieved by means of a bar-clip construction (Figure 3). The home care instructions regarding maintenance of the prosthesis and the soft tissues around the implants consisted of daily use of soap and water, along with mechanical cleaning of the abutments and connecting bar, using a soft toothbrush.

### Treatment outcome

After fabrication of the implant-retained auricular prosthesis, the patient was placed on a 12-month recall schedule. The clinical outcome parameters assessed included postoperative healing (inflammation and wound dehiscence), health of the peri-implant tissue, implant hygiene and loss of implants.

Postoperatively, no complications such as infection, adverse skin reactions or wound dehiscence occurred. The implant hygiene were good. No implants were lost.

There was a high degree of satisfaction with the auricular prosthesis. The patient reported no pain at all and did not report any frequent or permanent discomfort when wearing the conventional prosthesis.

## LITERATURE REVIEW

A complete search was conducted in the Medical Literature Analysis and Retrieval System Online (Medline) (1966 to May 2008), Literatura Latino-Americana e do Caribe em Ciências da Saúde (Lilacs) (1980 to May 2008) and Cochrane Center (1984 to May 2008) databases (Table 1). The following key words were used: ear, external (Medical Subject Headings/Descritores em Ciências da Saúde, MeSH/DeCS); and prostheses and implants (MeSH/DeCS). The reference lists of all the primary studies available were reviewed to identify any additional relevant citations. There were no language restrictions. All trials that seemed to be related on the basis of their titles, abstracts or MeSH headings were selected for full review.

Studies were further assessed for methodological quality with reference to the Oxford Centre for Evidence-Based Medicine Levels of Evidence Classification.^15^

**Table 1.** Search for studies about ear prostheses and implants in the literature

Database	Keywords	Result	Selected
PubMed	“Ear, External” [MeSH] AND “Prostheses and Implants” [MeSH]	19 clinical trials 127 case reports 0 meta-analyses 8 randomized controlled trials 624 unspecified	4 clinical trials 2 case reports
Lilacs	“Orelha Externa” [DeCS] AND “Próteses e Implantes” [DeCS]	2 articles	None
Cochrane Center	“Ear, External” [MeSH] AND “Prostheses and Implants” [MeSH]	0 articles	None

Lilacs = Literatura Latino-Americana e do Caribe em Ciências da Saúde; MeSH = Medical Subject Headings; DeCS = Descritores em Ciências da Saúde.

## INDICATIONS FOR BONE-ANCHORED AURICULAR PROSTHESES

A prosthetic device might be indicated in craniofacial reconstruction when plastic surgery is impossible or when the final cosmetic result is unsatisfactory (level of evidence, LE = 2b).^9^ The general indications for cranial implants with prosthetic reconstruction are lack of adequate tissue for reconstruction (LE = 4),^4^ absence of the lower half of the ear (LE = 2b),^16^ failed attempts at reconstruction (LE = 4),^4^ major cancer excision (LE = 2b),^16^ poor operative risks (LE = 2b)^16^ and selection of the technique by the patient (LE = 4).^4^ A fourth indication for prosthetic ear reconstruction is an acquired total or subtotal auricular defect, most often traumatic or ablative in origin, which is usually encountered in adults (LE = 2b).^16^ Prosthetic rehabilitation should be offered and discussed with such patients ahead of surgery and should be considered in particular for patients who reject multi-step reconstructive surgery or for whom this is not feasible (LE = 2b).^8^

Among pediatric patients, autogenous reconstruction is the procedure of choice. Prosthetic reconstruction of the auricle is considered for such pediatric patients if the following three relative indications apply: (i) failed autogenous reconstruction; (ii) severe soft-tissue/skeletal hypoplasia; and/or (iii) a low or unfavorable hairline (LE = 2b).^17^ Bone-anchored auricular prostheses are also indicated for treating severe congenital or acquired microtia in children, and the final result is generally very acceptable to the child (LE = 4).^18^

## ADVANTAGES AND DISADVANTAGES

The use of endosseous implants diminishes adhesive-related problems like discoloration and deterioration of the prosthetic material (LE = 2b).^19^ The skin and mucosal surfaces are less subject to mechanical irritation from intrinsic mechanical retention (LE = 3a)^20^ and chemical irritation from adhesives (LE = 4)^21^ or adhesive solvents (LE = 1c).^21^ In addition, the esthetics are enhanced because fine feathered margins can be maintained and positioning of an implant-retained craniofacial prosthesis is easier (LE = 4).^22^ Finally, from a clinical point of view, there are strong indications that implant-retained craniofacial prostheses have a positive impact on patients’ perceptions of such prostheses. In contrast with conventional craniofacial prostheses, implant-retained prostheses are often not experienced as prominent foreign objects in the head and neck region and may improve the quality of life (LE = 2b)^23^ - (LE = 4).^24^

Although the surgical techniques required for prosthetic reconstruction are less demanding than those for autogenous reconstruction are, construction of prostheses is a time-consuming task requiring experience and expertise. On the other hand, despite the technical challenge of autogenous reconstruction, prosthetic reconstruction requires lifelong attention and may be associated with late complications (LE = 2b).^16^

## SUCCESS RATE AND CLINICAL OUTCOME

Auricular osseointegrated implants have presented survival rates varying according to the length of follow-up, ranging from 92% after 8 years to 100% with shorter follow-ups (LE = 2b).^3^^,^^9^^,^^10^^,^^20^^,^^22^^,^^25^^,^^26^^,^^27^^,^^28^^,^^29^^,^^30^^,^^31^

Increasing age leads to greater failure of osseointegrated implants in the temporal bone. Blood flow in the temporal bone correlates well with patients’ ages, and this factor may be of importance for understanding why age influences implant survival (LE = 2b).^32^^,^^33^

Bone quality is also a critical factor in implant placement.^34^ Thus, craniofacial implant integration appears to be site-dependent (LE = 2b).^27^^,^^28^ Differences in volume and density could result in irradiation having a more destructive effect on the vascularity of this site, thereby compromising the potential for osseointegration (LE = 2b).^31^ The adverse biological changes that occur when osseous tissues are exposed to ionizing radiation include alterations in the cellular components of bone, involving significant reductions in the numbers of viable osteoblasts and osteocytes, as well as the development of areas of fatty degeneration within the bone marrow spaces. In addition, the blood vessels undergo progressive endarteritis, hyalinization and fibrosis, thus resulting in regional ischemia (LE = 2b).^27^^,^^32^

Several papers have raised concerns by describing significantly shorter survival rates when implants were placed in irradiated craniofacial bones, compared with non-irradiated sites (LE = 2b).^1^^,^^3^^,^^6^^,^^22^^,^^23^^,^^27^^,^^28^^,^^29^ Despite the well-documented adverse biological changes that occur when osseous tissues are exposed to ionizing radiation, craniofacial implants are now being placed with increasing frequency even in patients who have undergone irradiation, on the basis of the clinical success of such implants in the auricular area in particular. Notwithstanding the possible risks and disadvantages, it still seems reasonable to rehabilitate irradiated tumor patients with implants for craniofacial prostheses, while remembering that such patients who have undergone irradiation should be treated with caution. Hyperbaric oxygen (HBO) therapy can be used to improve the implant success rate, by 38% according to the literature (LE = 2b).^27^ However, in the mastoid region, HBO therapy might not be necessary before placement of the implants unless the patient has been irradiated with high radiation doses (LE = 2b).^28^

It is possible to place craniofacial implants in patients with oncological lesions of the head and neck during ablative surgery. Especially when radiotherapy is indicated, the possible advantages are the following: initial osseointegration takes place before irradiation and insertion of implants in a compromised area can be avoided; earlier prosthetic rehabilitation; and surgical intervention in irradiated tissue is limited to second-stage surgery (LE = 2b).^35^ However, there is general concern among head and neck surgeons and radiotherapists that metal implants within the irradiated field may, because of scattering, cause an overdose in the adjacent tissue over the course of radiation therapy. This could lead to three consequences: 1) smaller irradiation dose reaching the tumor if it is situated behind the implants; 2) possible loss of osseointegration and implant failure because of the higher irradiation dose; and 3) increased risk of osteoradionecrosis developing in the bone adjacent to the implant (LE = 2b).^35^ To avoid these potential complications, it is recommended that all unnecessary metal objects are avoided in the radiation field. Thus, fixtures are installed at the time of ablative surgery and the tumor bed is irradiated. After the acute clinical reactions have declined, second-stage implant surgery is performed, abutments and retention elements are connected and the prostheses are constructed. Currently, bars with retention clips are favored for auricular prostheses (LE = 2b).^35^^,^^36^

The skin penetration site is the single factor that has caused the most significant clinical problems regarding craniofacial osseointegration. Despite extensive subcutaneous reduction during surgery, some patients will experience reddened and moistened skin, and sometimes granulation tissue forms around the abutment (LE = 2b).^24^ The skin around the implants must be cared for regularly by the patients with daily cleansing (LE = 3b),^37^ combined with adjustments by the clinician at follow-up patient visits (LE = 2b).^14^

The frequency of adverse skin reactions around the soft tissues of the percutaneous implant is generally very low (LE = 2b).^5^^,^^9^^,^^13^^,^^14^^,^^38^ The main symptomatic reactions may consist of slight redness, reddened and moistened peri-implant tissues, granulation tissue associated with the implants or infection of the peri-implant soft tissues (LE = 2b).^5^^,^^9^^,^^13^^,^^14^ Good patient hygiene compliance combined with thin and immobile peri-implant soft tissues have been found to result in minimal soft tissue complications (LE = 2b).^5^^,^^9^^,^^13^^,^^14^ The frequency and degree of adverse skin reactions have been seen to decrease with time (LE = 2b).^5^^,^^9^^,^^13^^,^^14^^,^^38^ Young patients have higher incidence of adverse skin reactions (LE = 2b).^31^^,^^35^ The likelihood of losing an implant because of adverse skin reactions is quite low, but if these skin reactions are left untreated, implant loss or withdrawal may eventually occur (LE = 2b).^39^

The cosmetic results and patient acceptance have been very satisfactory (LE = 2b),^40^^,^^41^ with few postoperative complications. Most patients have found that caring for the skin around the abutments did not cause any notable problems (LE = 2b).^42^

## FINAL CONSIDERATIONS

The surgical technique for auricular prostheses retained on osseointegrated implants seems to be simple and is associated with a low rate of perioperative and long-term complications.

The major advantages of this technique are that it puts less strain on the patient and has superior esthetics, compared with traditional surgical reconstructive techniques. The disadvantages of the method are the lifelong daily skin care and dependence on the health services that are required.

Radiotherapy is not a contraindication for the use of osseointegrated implants in the maxillofacial region, but the loss of implants is higher in irradiated sites than in non-irradiated sites.

Bone-anchored titanium implants may provide patients with a safe and reliable method for anchoring auricular prostheses that enables restoration of their normal appearance and offer an improvement in their quality of life. Hence, the use of bone-anchored prostheses should be considered to be a viable alternative to surgical reconstruction.
